# Radiological assessment of irrigation water used in Rustenburg, South Africa

**DOI:** 10.1093/rpd/ncad080

**Published:** 2023-05-24

**Authors:** Oluwadamilare P Olagbaju, Olarenwaju B Wojuola, Makondelele V Tshivhase

**Affiliations:** Physics Department, North-West University, South Africa; Physics Department, North-West University, South Africa; Physics Department, University of Johannesburg, South Africa

## Abstract

Water is an essential input in agricultural production, which is why it plays an important role in food security. According to the World Bank, water irrigated agriculture represents about 20% of the total cultivated land and 40% of the total food produced globally. This makes water a direct and indirect route of radiation exposure to humans via contact, ingestion and consumption of agricultural products. Radiological assessment of irrigation water around Rustenburg, one of the mining and industrial cities in South Africa, is investigated in this study. The activity concentrations of ^238^U, ^232^Th and ^40^K in irrigation water samples were determined using the total mass elemental concentrations of uranium, thorium and potassium, measured using inductively coupled plasma mass spectroscopy. The activity concentrations of ^238^U and ^40^K range from 1.24 × 10^−04^ to 1.09 × 10^−02^ Bq/l, and 7.07 × 10^00^ to 1.32 × 10^+01^ Bq/l, with mean activity concentrations of 2.78 × 10^−03^ and 1.16 × 10^+01^ Bq/l, respectively. The activity concentration of ^232^Th was found below the detection level in all sampled irrigation water. Estimated annual effective dose because of ingestion because of ^238^U and ^40^K was also found to be below 120 μSv/y for ^238^U and ^232^Th, 170 μSv/y for ^40^K and a total of 290 μSv/y by the United Nations Scientific Committee on the Effects of Atomic Radiation. The estimated radiation dose and lifetime cancer risk indices indicate insignificant radiological risk, making the irrigation water safe for domestic and agriculture purposes.

## Introduction

Environmental pollution is a global concern because of the numerous mining, industrial and domestic wastes discharged into the terrestrial and aquatic system, and the innumerable health challenge they present^(1)^. Radionuclides are naturally present in environmental media resulting from rock and mineral weathering^(2)^. Their concentration in the environment is enhanced by contributions from various anthropogenic sources such as nuclear, medical, agricultural and industrial operations, making it vary from region to region, depending on each region's geological and anthropogenic condition.

In mining, radioactive isotopes are produced along with radioactive dust and transported through groundwater flow and wind to agricultural lands and water bodies, thereby serving as sources of exposure to humans via contaminated food and water^(3, 4)^. Radioactive nuclides, though used in human medicines, radioactive dating and research, are widely known for their carcinogenic and noncarcinogenic health effects^(2)^.

Global attention, given to the safety of agricultural produce because of the role they play in the human diet, has led to the investigation of agricultural lands and irrigation water through which they find their way into edible plant parts, especially in regions with potential sources of radionuclides^(5, 6)^. According to Adesiyan *et al*.^(3)^, naturally occurring radionuclides in surface and groundwater result in drinking and irrigation water contamination, posing a significant risk to human health via various exposure pathways.

South Africa, which has one of the most diverse and comprehensive farming systems in Africa, is known for its mining and industrial activities, resulting in radioactive waste littered in farmlands and communities^(7)^. South Africa is a water-scarce country, having about 12% of the country’s land suitable for growing rain-fed crops with 3% considered fertile, and about 1.3 million ha of land under irrigation, which represents 60% of the total water use per sector, according to Baleta and Pegram^(8)^, contributing significantly to the food chain, which serves as direct exposure source through ingestion of food crop. This article is aimed to determine the activity concentration of naturally occurring radionuclides in irrigation water and its associated human health risk.

### Sampling

About 1-l water sample was collected in polyethene bottles from 17 irrigation water sources, ranging from artificial dams, ponds, in the farming area of Rustenburg. The used polyethene bottles were thoroughly washed, and 10 ml of HCl was added to sampled water immediately after collection to avoid radiotoxic absorption on the container walls^(9)^. The coordinates of each sampling location were recorded using a Global Positioning System to ensure traceability. All the samples were well labelled for ease of identification.

### Methodology

Inductively coupled plasma-mass spectrometry (ICP-MS), which consists of samples introduction system and data processing unit, ions source, the mass analyser and the detector, was used. The total quant method was adopted to analyse digested samples because of its high sensitivity, wide linear dynamic detection range and simultaneous multielement analysis^(10)^. ICP-MS calibration is done by measuring the instrumental response to reference standard solution (a 10 mg/l multielement calibration standards Ag, Al, As, Ba, Be, Bi, Ca, Cd, Co, Cr, Cs, Cu, Fe, Ga, In, K, Li, Mg, Mn, Ne, Ni, Pb, Rb, Se, Sr, Tl, U, V and Zn).

The filtrates from digested samples were analysed in triplicate using ICP-MS. For every eight samples measured, the instrument is set to run a certified standard prepared in the same acid matrix and blank solution for quality control and to ensure there's no drift and cross-contamination of samples^(11)^. The elemental concentration of heavy metal in mg/l was estimated using the expression ^(12)^


(1) \begin{equation*} Concentration\left(\frac{\mu g}{g}\right)=C\times \frac{V_f}{w\times s}\times \frac{DF}{1000} \end{equation*}

where *C* = instrument value in 11 g/l (the average of all replicate integrations), *V_f_* = final digestion volume (ml), *W* = initial aliquot amount (g), *S* = % solids/100.

Elemental concentration of naturally occurring radionuclide (ppb) measured using ICP-MS was converted into activity concentration (Bq/kg) using ‘ppm to Bq/kg’ conversion factors of ^238^U—1 ppm = 12.35 Bq/kg; ^232^Th—1 ppm = 4.06 Bq/kg and 1% of ^40^K = 313 Bq/kg, given by International Atomic Energy Agency ^(13, 14)^.

**Table 1:** Activity concentration of naturally occurring radionuclides in irrigation water.

Sample ID	^238^U (Bq/l)	^40^K (Bq/l)
W01	5.80 × 10^−03^	1.29 × 10^+01^
W02	1.09 × 10^−02^	1.24 × 10^+01^
W03	9.88 × 10^−04^	1.26 × 10^+01^
W04	5.93 × 10^−03^	1.25 × 10^+01^
W05	5.06 × 10^−03^	1.32 × 10^+01^
W06	3.83 × 10^−03^	1.13 × 10^+01^
W07	4.69 × 10^−03^	1.32 × 10^+01^
W08	3.21 × 10^−03^	1.14 × 10^+01^
W09	1.11 × 10^−03^	1.14 × 10^+01^
W10	9.88 × 10^−04^	1.16 × 10^+01^
W11	1.11 × 10^−03^	8.51 × 10^+00^
W12	8.65 × 10^−04^	1.32 × 10^+01^
W13	7.41 × 10^−04^	1.27 × 10^+01^
W14	8.65 × 10^−04^	1.30 × 10^+01^
W15	8.65 × 10^−04^	1.23 × 10^+01^
W16	1.24 × 10^−04^	7.07 × 10^+00^
W17	2.47 × 10^−04^	8.04 × 10^+00^
Maximum	1.09 × 10^−02^	1.32 × 10^+01^
Minimum	1.24 × 10^−04^	7.07 × 10^+00^
Average	2.77 × 10^−03^	1.16 × 10^+01^

**Table 2:** Ingestion dose of naturally occurring radionuclides in irrigation water.

Sample ID	^238^U (mSv/y)	^40^K (mSv/y)	Total (mSv/y)	LCR
W01	1.57 × 10^−07^	4.79 × 10^−05^	4.81 × 10^−05^	1.68 × 10^−04^
W02	2.93 × 10^−07^	4.60 × 10^−05^	4.63 × 10^−05^	1.62 × 10^−04^
W03	2.67 × 10^−08^	4.70 × 10^−05^	4.70 × 10^−05^	1.64 × 10^−04^
W04	1.60 × 10^−07^	4.64 × 10^−05^	4.66 × 10^−05^	1.63 × 10^−04^
W05	1.37 × 10^−07^	4.93 × 10^−05^	4.94 × 10^−05^	1.73 × 10^−04^
W06	1.03 × 10^−07^	4.21 × 10^−05^	4.22 × 10^−05^	1.48 × 10^−04^
W07	1.27 × 10^−07^	4.89 × 10^−05^	4.91 × 10^−05^	1.72 × 10^−04^
W08	8.67 × 10^−08^	4.24 × 10^−05^	4.25 × 10^−05^	1.49 × 10^−04^
W09	3.00 × 10^−08^	4.26 × 10^−05^	4.26 × 10^−05^	1.49 × 10^−04^
W10	2.67 × 10^−08^	4.33 × 10^−05^	4.33 × 10^−05^	1.52 × 10^−04^
W11	3.00 × 10^−08^	3.17 × 10^−05^	3.17 × 10^−05^	1.11 × 10^−04^
W12	2.33 × 10^−08^	4.93 × 10^−05^	4.93 × 10^−05^	1.73 × 10^−04^
W13	2.00 × 10^−08^	4.71 × 10^−05^	4.72 × 10^−05^	1.65 × 10^−04^
W14	2.33 × 10^−08^	4.84 × 10^−05^	4.84 × 10^−05^	1.69 × 10^−04^
W15	2.33 × 10^−08^	4.59 × 10^−05^	4.59 × 10^−05^	1.61 × 10^−04^
W16	3.33 × 10^−09^	2.63 × 10^−05^	2.63 × 10^−05^	9.21 × 10^−05^
W17	6.67 × 10^−09^	2.99 × 10^−05^	2.99 × 10^−05^	1.05 × 10^−04^
Maximum	2.93 × 10^−07^	4.93 × 10^−05^	4.94 × 10^−05^	1.73 × 10^−04^
Minimum	3.33 × 10^−09^	2.63 × 10^−05^	2.63 × 10^−05^	9.21 × 10^−05^
Average	7.51 × 10^−08^	4.32 × 10^−05^	4.33 × 10^−05^	1.51 × 10^−04^

The ingestion dose *E_ing_* (mSv/y) from the consumption of ^238^U, ^232^Th and ^40^K in water was estimated using the following equation^(15)^: 


(2) \begin{equation*} {E}_{ing}\left( mSv/ Yr\right)={C}_{\tau }{I}_{ing}\sum \limits_{j=1}^3 DC{F}_{ing} \end{equation*}

where *C_τ_* is the activity concentration of the radionuclides in a sample, *L_ing_* is the consumption rate per year, *DCF_ing_* = the effective dose coefficient in Sv/Bq for the ingestion of natural radionuclides.

Effective dose coefficient of 4.50 × 10^−8^ Sv/Bq, 2.30 × 10–7 Sv/Bq, 6.20 × 10^−9^ Sv/Bq was used for ^238^U, ^232^Th and ^40^K, respectively^(16)^. The average consumption rate of 600 l/y was used to estimate annual effective doses in sampled water^(17)^.

Lifetime cancer risk (LCR) was calculated using equation (3), where *E_ing_* is the ingestion dose (Sv y^−1^) from the consumption of ^238^U, ^232^Th and ^40^K in water; LE is the life expectancy (70 y); and RF is the risk factor (Sv^−1^). Recommended nominal probability coefficient for radiation-induced stochastic health effects of 7.3 × 10^−2^ Sv^−1^ was used for risk assessment:


(3) \begin{equation*} LR={E}_{ing}\times LE\times RF \end{equation*}

## Results and discussion

The activity concentrations of naturally occurring radionuclides in water samples collected from dams used for irrigation in the Rustenburg area, South Africa, are presented in Table 1. The activity concentrations of ^238^U and ^40^K range from below detection limit to 1.09 × 10^−02^ Bq/l, and 7.07 × 10^00^ to 1.32 × 10^+01^ Bq/l, with a mean activity concentration of 2.77 × 10^−03^and 1.16 × 10^+01^ Bq/l, respectively, whereas ^232^Th was not detected in all measured water samples. A similar observation was made by Ugbede and Osahon^(18)^. The results imply that the sampled irrigation water is fit for human use and agricultural purposes because elemental concentration of uranium and thorium is below the World Health Organization and United States Environmental Protection Agency’s guidelines (15 and 30 μg/l, respectively).

Table 2 presents the ingestion dose resulting from intake of irrigation water and its associated excess LCR. The average committed effective dose because of ingestion because of ^238^U and ^40^K in irrigation water is 7.51 × 10^−08^ and 4.32 × 10^−05^ mSv/y, respectively. They are below the reported global average of 2.4 mSv/y^(15, 19)^. Similarly, excess LCR is found to be less than the global average of 2.5 × 10^−3^ reported by the United Nations Scientific Committee on the Effects of Atomic Radiation. This indicates an insignificant radiological risk, making the irrigation water safe in the study area for domestic and agriculture purposes.

## Conclusion

The activity concentration of ^238^U, ^232^Th, ^40^K in irrigation water collected from dams and pounds was measured to assess the radiological risk because of the ingestion of naturally occurring radionuclides for a mining area. The results show that average committed effective dose because of ingestion of naturally occurring radionuclides was below the dose recommended by the UNSCEAR (2000). Also, the average LCR resulting from the ingestion of naturally occurring radionuclides in water was significantly < 2.5 × 10^−3^ based on an annual effective dose limit of 1 mSv for the general population, reported by the International Commission on Radiological Protection. The results obtained indicate an insignificant radiological risk, making the irrigation water of the study area safe for domestic and agriculture purposes. Continuous radiological monitoring is recommended because of continuous mining activities and the use of agrochemicals in the study area.
